# Application of *Ficus carica* L. and *Solanum incanum* L. Extracts in Coagulation of Milk: The Case of Traditional Practice in Ab'ala Area, Afar Regional State, Ethiopia

**DOI:** 10.1155/2020/9874949

**Published:** 2020-04-07

**Authors:** Welday Desta, Mohammed Shumbahri, Sibhatu Gebrehiwot

**Affiliations:** ^1^Department of Chemistry, Samara University, Semera, Afar Region, Ethiopia; ^2^Department of Biology, Samara University, Semera, Afar Region, Ethiopia; ^3^Raya University, Maichew, Tigray Region, Ethiopia

## Abstract

People living in and around Ab'ala area of the Afar Regional State, Ethiopia, have a traditional practice of applying *Ficus carica* leaf and *Solanum incanum* fruit extracts to milk in order to coagulate it as soon as possible. Thus, to investigate the role of the extracts in the coagulation of milk and their health threats, the milk-clotting activity, phytochemical screening tests, antimicrobial activities by the agar well diffusion method, and heavy metal content by ICP-OES technique were determined. Accordingly, both *Ficus carica* and *Solanum incanum* were found to possess phenolics, saponins, and tannins. Likewise, positive tests for flavonoid in *Ficus carica* and alkaloid in *Solanum incanum* were observed. However, no terpenoids, glycosides, and oxalates were detected in the plants. Moreover, the crude and concentrated enzyme extracts of the plants exhibited clotting activity. In this regard, the enzyme extracts of *Ficus carica* were superior with the highest clotting activity of 1.20 U. On the other hand, ethanol and chloroform extracts of the samples showed inhibition zones against all tested microorganisms except their chloroform extract which did not exhibit inhibition against *Escherichia coli* and *Aspergillus niger*. Likewise, the metals Cr, Cd, Mn, Cu, and Fe were detected in the plant samples, with the Mn content of 3.67 ± 0.10 mg per kg of dry weight of the plant in *Ficus carica* being the highest. Indeed, the level of the heavy metal contents is considerably lower than those maximum permissible limits set by international standards. On the other hand, no Pb and Zn were detected in the plant samples. Therefore, the higher clotting activity of the enzyme extracts was an indicator that enzymes, rather than other phytochemicals, are the most probable agents responsible for the milk-clotting ability of the plants, resulting in the formation of cheese. Furthermore, the growth inhibition to most of the test microbes is a manifestation that bacterial fermentation is not a means of clotting the milk as bacteria introduced to the milk would be killed by the sample extracts. Moreover, the use of the plants in the coagulation process would not pose health threats as far as oxalate and metal toxicity is concerned.

## 1. Introduction

Africa is well known for its floral biodiversity, exploited by indigenous people for traditional medicinal purposes. However, adequate studies have not been done to examine the existence of bioactive compounds and possible biological properties of the medicinal plants [1, 2]. Promising results have been obtained in the emerging research investigations on the therapeutic potentials of African flora as reviewed by Gurib-Fakim and Mahomoodally [3]. Moreover, plants are known for their use as spices applied to modify the flavor and aroma of foods and beverages depending on the knowledge and tradition of local practitioners. One of the customary uses of plants is in milk coagulation. There are many alternatives such as heating and adding acidic compounds (such as phenolic phytochemicals), well-cultured microbials, and enzymes that convert milk into coagulant products such as yogurt and cheese. The coagulation of milk by enzymatic methods is a basic step in the manufacture of most cheeses. Chymosin is the principal milk-clotting protease present in the natural calf rennet and has been used for centuries as an aid in the cheese-making process [4]. On the other hand, in dairy farms, well-cultured bacteria are used to make yogurt and the two bacteria most commonly added to milk are *Lactobacillus bulgaricus* and *Streptococcus thermophiles* [5].

Although the coagulation process of milk may be achieved through time by fermentation, obtaining coagulated milk products such as yogurt and cheese as soon as the milk is obtained is hard, especially in the cold seasons. Since it is difficult to get coagulating agents such as enzymes and bacteria at household levels, the people of Ab'ala area instead developed a tradition of using *Ficus carica* and *Solanum incanum* plant extracts found in their natural environment. Although the use of these plants in the specified area is mostly practiced in the assumption of facilitating the formation of yogurt, there is also a possibility of forming cheese as plants are able to release enzymes which act as that of chymosin [6]. The thorough formation of yogurt can be possible only if the plant extracts support fast bacterial growth that can act like *Lactobacillus bulgaricus* and *Streptococcus thermophilus*. If this is not the case, the formation of cheese is most likely and this does not give quality milk products such as butter that are formed with the formation of yogurt.

On the other hand, although plant extracts are widely considered to pose less risk than synthetic drugs and food additives, they are not completely free of toxic or other side effects. It is known that plants readily assimilate metal elements through their roots. Despite the nutritive value of most of these elements at lower concentration, some metals are toxic even at very low concentration [7]. Because of the possibility of accumulation of toxic metals in plants, there is an increasing interest in the determination of their contents. Moreover, there are phytochemicals called oxalic acids and their salts are known to pose kidney dysfunction due to their ability to bind with some metal ions forming a condition called kidney stone. When plants rich in these compounds are utilized as food sources or ingredients, they may have adverse health effects [8]. Thus, it is imperative to monitor the level of toxic constituents in plants while using them as food supplements.

To the best of our knowledge, no study has been conducted to scientifically examine the role of *Ficus carica* and *Solanum incanum* plants in the process of coagulating milk. Thus, the main purpose of the study was to investigate the clotting agents—whether phytochemicals, enzymes, and/or microbials—in order to understand the function of the extracts in the coagulation process of milk and identify the most likely product—whether cheese or yogurt. Moreover, since crude extracts contain a mixture of many substances, the presence of selected heavy metals (Pb, Cr, Cd, Mn, Cu, Zn, and Fe) and oxalates was assessed in order to alert the users to the probable consequences of utilizing these plants.

## 2. Materials and Methods

### 2.1. Collection and Preparation of Samples

*Ficus carica* leaf and *Solanum incanum* fruits were collected from Ab'ala town area and were first washed with distilled water in order to avoid dusts and contaminants due to handling during collection. Then, they were air-dried in order to remove surface moisture. Next, each sample was sliced into pieces of equal size and placed in an oven at 40°C for further drying until constant mass was obtained. Finally, the dried samples were pulverized into powder using an electronic mixer grinder, stored in polyethylene bag, and reserved for further analysis.

### 2.2. Phytochemical Screening Tests

In investigating the phytochemical composition of the plants, 5.00 g of each sample powder was extracted using 100 mL of ethanol. Then, the mixture was filtered with Whatman No. 1 filter paper, and the filtrate was tested for the presence of phenolic compounds, saponins, oxalates [9], flavonoids [10], alkaloids, terpenoids, tannins [11], and glycosides [12].

### 2.3. Determination of Milk-Clotting Activity

To determine the milk-clotting activity of the plant extracts, 10.00 g of each sample powder was extracted using 200 mL of distilled water. Then, the mixture was filtered with Whatman No. 1 filter paper. This filtrate was designated as “crude extract” and was kept for further analysis. Meanwhile, concentrated enzyme extract was obtained by taking 100 mL of the already prepared crude extract and precipitated with solid ammonium sulphate at 80% (*w*/*v*) saturation. This content was allowed to stand in ice bath for 30 minutes. The resulting precipitate was dissolved in twice its volume with 0.02 M phosphate buffer (pH = 6). This solution was filtered with Whatman No. 1 filter paper to remove any solid particles [13]. Milk-clotting activities of the crude and enzyme fractions were then determined as described by Abdalla et al. [14] with some modifications. And 10 mL of assay milk [10% (*w*/*v*) skimmed milk powder in distilled water] was taken in a test tube and the content was brought to 37°C through heating in a water bath. 2.0 mL of each crude and enzyme extract was added and the curd formation was observed while manually rotating the test tube with tilting at some time interval. One milk-clotting unit (U) is defined as the amount of extract clotting 10 mL of milk substrate in 5 min at 37°C, and it was obtained by the following equation:

(1) $$\begin{array}{c} \text{milk}-\text{clotting activity}\left(\text{U}\right)=\frac{\text{volume of extract}}{\text{clotting time}\left(\min\right)}\times 5. \end{array}$$

Each analysis was done in triplicate and 2.0 mL of distilled water for the crude extract and 2.0 mL solution of ammonium sulphate (80%, *w*/*v*) in 0.02 M phosphate buffer (pH = 6) for the enzyme extract were used instead of the respective sample extracts as a control.

### 2.4. Preparation of Ethanol and Chloroform Extracts

Ethanol and chloroform were used as extracting solvents. Accordingly, 50.00 g of each sample powder was extracted with 200 mL of the respective solvents. Then, the mixture was kept in a conical flask and was shaken in a rotary shaker at 121 rpm for 24 hrs. to ensure thorough mixing and enough maceration of the plant parts. After 24 hrs., the suspension was filtered separately with Whatman No. 1 filter paper. The resulting filtrate was concentrated using a vacuum rotary evaporator at 40°C to confiscate the solvent. After the evaporation of solvents, the residual crude extracts were weighed and recorded. The extracts were kept in a refrigerator at 4°C for further work [15].

### 2.5. Determination of Antibacterial Activity

Agar well diffusion method was used to test the antibacterial activity of the sample extracts. A well-cultured bacterial suspension (compared visually with 0.5 McFarland turbidity standard) of four microorganisms (*Bacillus subtilis, Shigella flexneri, Escherichia coli*, and *Ralstonia solanacearum*) were seeded in a Petri dish containing freshly prepared Mueller–Hinton agar medium. Then, agar wells were prepared using a cork borer and the extracts were applied, in triplicate, to the wells. An antibiotic drug (amoxicillin) was used for comparison as a positive control and the solvent was used as a negative control. Next, these contents were incubated at 37°C for 24 hours. The diameters of inhibition zones (clear transparent regions), including the well diameter, are reported in mm [16].

### 2.6. Determination of Antifungal Activity

The same sample extraction procedure as that of the antibacterial activity was used for this determination and the agar well diffusion method was used to test the antifungal activity of the sample extracts. Based on this, two well-diluted fungal cultures (*Aspergillus niger* and *Penicillium cyclopium*) were seeded into a Petri dish containing freshly prepared potato dextrose agar. Agar wells were prepared using a cork borer, and the sample extracts were added into the wells. Then, these contents were incubated at 30°C for 48 hours and observed for inhibition of fungal growth. The diameters of inhibition zones (clear transparent regions), including the well diameter, are reported in mm [16].

### 2.7. Determination of Heavy Metal Content

#### 2.7.1. Microwave-Assisted Acid Digestion

The microwave-assisted acid digestion was used based on prior method [17]. About 1.0 g of each sample was digested with 6 mL of HNO_3_ and 2 mL of H_2_O_2_ in microwave digestion system by setting the operating parameters as presented in Table 1. The resulting solutions were cooled, transferred into a 50 mL vial, and diluted with distilled water to the mark. These contents were then analyzed by the inductively coupled plasma-optical emission spectrophotometer (ICP-OES).

#### 2.7.2. ICP-OES Determination

ICP-OES 5100 Synchronous Vertical Dual View (Agilent Technologies, USA) spectrophotometer was applied for the determination of metals using a standard calibration method. The operating conditions employed for ICP-OES determination are tabulated in Table 2. This method was employed based on the procedures described by Bizzi et al. [18] with some modifications. One mL of each of the microwave-digested sample solutions was pumped through a nebulizer into a spray chamber. The produced aerosol was led into argon plasma leading to an excited state of the samples. The excited atom emits a radiation (*s*) when it goes back to ground state. The emitted characteristic radiations and intensities were measured optically by detectors. Amount of light absorbed was proportional to the concentration of the metal excited with respect to the selected wavelength.

The wavelengths selected for the ICP-OES determination of the monitored elements are given in Table 3. The amount of the metals present was determined from a calibration curve of absorbance versus concentration of standard solutions of the respective metals. The calibration standards were prepared by diluting the stock multielemental standard solution (1000 mgL^−1^) in 0.5% (*v*/*v*) nitric acid. The calibration curves for all the studied elements were in the range of 0.01 to 1.0 mgL^−1^.

### 2.8. Method of Statistical Analysis

The data were based on three replicates and subjected to one-way analysis of variance (ANOVA). Standard errors of each individual extract of the samples when applied to the determination of antimicrobial and heavy metals were computed, and variations were evaluated by least significant difference (LSD) at 5% level of probability (*p* < 0.05). Data analysis was conducted using the SPSS 20 Statistical Software Package.

## 3. Results and Discussion

### 3.1. Phytochemical Screening Tests

The presence of phenolic compounds, flavonoids, alkaloids, saponins, terpenoids, glycosides, tannins, and oxalates was investigated in the sample extracts by different test methods. Both *Ficus carica* and *Solanum incanum* exhibited positive results for phenolics, saponins, and tannins. On the other hand, flavonoids were found only in *Ficus carica*, while alkaloids were found in *Solanum incanum*. However, terpenoids, glycosides, and oxalates were not detected on the extracts of both plants (Table 4). Similar results were reported by other studies on *F. carica* [19, 20] and *S. incanum* [21–27], respectively. However, Desta and Haftom had reported findings contrary to the present study in which they detected terpenoid in extract of *S. incanum* [28].

### 3.2. Milk-Clotting Activity

Rennet is a term applied to any crude enzyme preparation of animal, plant, or microbial origin which curdles milk. Plant enzymes are relatively safe, inexpensive, readily available and are generally acceptable for such applications [29]. *Ficus carica* and *Solanum incanum* are among the plant families whose crude and enzyme extracts can be applied to milk coagulation (Table 5).

In this regard, the enzyme extract of *Ficus carica* was the most promising coagulant with highest milk-clotting activity of 1.20 U followed by the enzyme extract of *Solanum incanum* as shown in Table 5. This is evident that the coagulating agents responsible for the clotting process are enzymes obtained from the plant extracts rather than other phytochemical components.

### 3.3. Antimicrobial Activities

**Figure 1:**
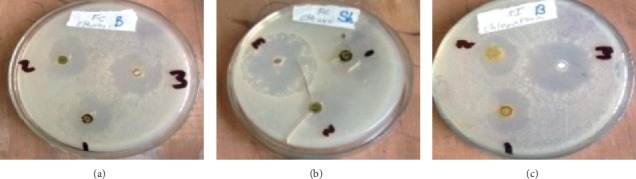
Inhibition zones (in mm) of (a) *Ficus carica* and AML drug against *Bacillus subtilis*, (b) *Ficus carica* and AML drug against *Shigella flexneri*, and (c) *Solanum incanum* and AML drug against *Bacillus subtilis* bacteria.

The antibacterial activity of sample extracts was determined by the agar well diffusion method. After applying the extract solutions into the wells of the inoculum and incubating for 24 hrs. at 37°C, the formation of inhibition zones (clear transparent regions) around the wells (Figure 1) was observed.

From Table 6, it can be observed that the entire sample extracts show certain bioactivity against the selected test microorganisms except their chloroform extract which does not show inhibition against *Escherichia coli*. The fact that this sample extracts showed inhibition zones against such microorganisms justifies that bacteria cannot be a coagulating agent within the coagulation time. This is because the growth of introduced bacteria will be inhibited by the sample extracts before it becomes active to help milk fermentation. Rather, the sample extracts are important in keeping the milk safe by prohibiting microbes introduced due to hygienic problems during the coagulation process. Studies reported by others [25, 28] showed compliment results with the present study in regard to the antibacterial activity of *Solanum incanum.* Likewise, Jeong et al. [30] had reported findings comparable to ours, where *Ficus carica* had profound antimicrobial activities against tested bacteria.

The antifungal activity of the sample extracts was also determined by the agar well diffusion method. After applying the extract solutions into the wells of the inoculum and incubating for 48 hrs. at 30°C, the formation of inhibition zones (clear transparent regions) around the wells was observed and the corresponding values are tabulated in Table 7. From Table 7, all the extracts show antifungal activity except the chloroform extract of *Ficus carica* and *Solanum incanum* which do not show inhibition against the growth of *Aspergillus niger* fungus. On the other hand, these same extracts showed the highest antifungal activity against *Penicillium cyclopium* fungus. This may be considered as the advantage of the plants in prohibiting fungal growth during the milk processing stages.

The finding that the studied plants showed antimicrobial activities can be used as significant evidence that the use of the plants to coagulate milk most likely leads to the formation of cheese rather than yogurt. However, the possibility of yogurt formation should be ruled out through further study of the quality aspects of the coagulum formed.

### 3.4. Heavy Metal Content

The contents of lead (Pb), chromium (Cr), cadmium (Cd), manganese (Mn), copper (Cu), zinc (Zn), and iron (Fe) metals in *Ficus carica* and *Solanum incanum* samples were determined using ICP-OES, and the measured concentrations of metal elements in the respective plant materials are reported in Table 8.

Among the considered metal contents, the Mn content in *Ficus carica* was the highest. On the other hand, Pb and Zn metals were not detected in the sample solutions and the concentration of Cd—one of the known toxic metals—was remarkably the lowest in both sample plants. Moreover, the contents of the metals in the plants were below the maximum permissible limit set by WHO for medicinal plants.

Since soil is the main feed of nutrients for plants, increasing the heavy metal content in soil also increases the uptake of heavy metals by plants. Meanwhile, the most important sources of heavy metals in the soil are the anthropogenic activities such as mining, smelting procedures, steel and iron industry, chemical industry, traffic, and agriculture as well as domestic activities. The fact that the metal levels of the studied plants are considerably low may be an indicator that the practice of such human activities is insignificant in the study site.

## 4. Conclusion

The extracts of *Ficus carica* and *Solanum incanum* showed milk-clotting activity which might most likely be due to an enzyme component. On the other hand, the same extracts showed antibacterial activity which is an indicator that bacteria cannot be responsible for the clotting of milk in the presence of the plant extracts. This leads to the inference that the product of the milk-clotting process due to the application of the studied plant extracts is most likely cheese. However, ruling out the possibility of yogurt formation needs further study regarding the quality aspects of the coagulum formed. Meanwhile, the absence of phytochemicals called oxalates and the low level of heavy metals in the plants compared to the maximum permissible limits set by international standards such as WHO may indicate the fact that the use of such plants does not considerably pose health risks as far as oxalate and metal toxicity is concerned. The present study shows that the use of such plants leads to the most probable formation of cheese rather than yogurt. Thus, further study is recommended to study factors such as taste- and texture-related quality aspects of the coagulum in order to investigate whether it fulfills the quality of yogurt or not. Moreover, since test tube (*in vitro*) reactions are very much different from reactions in the cell (*in vivo*), the biochemical profile of the plant extracts should be studied to be recommended as completely safe for human use.

## Figures and Tables

**Table 1:** Operating conditions of microwave digestion.

Parameters	Stage			
	1	2	3	4
Temperature	160	120	100	100
Ramp time/min	5	1	1	1
Hold time/min	25	5	1	1
Power/W	840	280	140	140

**Table 2:** Operational conditions for ICP-OES.

Parameter	Description
Radio-frequency power (kW)	1.3
Plasma gas flow rate (Lmin^−1^)	15.0
Auxiliary gas flow rate (Lmin^−1^)	2.0
Nebulizer gas flow rate (Lmin^−1^)	0.80
Spray chamber	Scott double-path type
Nebulizer	Cross flow
Observation view	Axial

**Table 3:** List of wavelengths selected for the respective metals.

Metal	Wavelength (nm)
Lead (Pb)	220.3
Chromium (Cr)	367.7
Cadmium (Cd)	214.4
Manganese (Mn)	279.8
Copper (Cu)	324.7
Zinc (Zn)	213.9
Iron (Fe)	259.9

**Table 4:** Phytochemical screening of *S. incanum and Ficus carica* crude extracts.

S. no.	Phytochemicals	*S. incanum*	*Ficus carica*
1	Alkaloids	+	−
2	Saponins	+	+
3	Flavonoids	−	+
4	Glycosides	−	−
5	Terpenoids	−	−
6	Tannins	+	+
7	Oxalates	−	−
8	Phenolics	+	+

“+” indicates the presence of the tested phytochemical. “−” indicates the absence of the tested phytochemical.

**Table 5:** Clotting activity of sample extract.

Sample extract		Volume of extract (mL)	Clotting time (min)	Clotting activity (U)
*Ficus carica*	Crude	2.0	12.4±0.1^*a*^	0.81±0.01^*a*^
	Enzyme	2.0	8.3±0.2^*b*^	1.20±0.03^*b*^
*Solanum incanum*	Crude	2.0	16.3±0.2^*c*^	0.61±0.01^*c*^
	Enzyme	2.0	10.4±0.3^*d*^	0.90±0.03^*d*^
Control		2.0	245.0^*e*^	—

Data are expressed as a mean of three determinations ± SD. There is a significant difference in the clotting times and clotting activities with different-letter superscripts down a column (*p* < 0.05).

**Table 6:** Inhibition zones, including well diameter, of samples against bacterial growth.

Solvent	Samples and antibiotic drug	Inhibition zones (in mm) for bacteria			
		*Bacillus subtilis*	*Shigella flexneri*	*Escherichia coli*	*Ralstonia solanacearum*
Ethanol	*Ficus carica*	^(1)^11.87±0.11^*a*^	^(1)^11.73±0.11^*a*^	^(1)^9.13±0.11^*b*^	^(1)^8.07±0.11^*c*^
	*Solanum incanum*	^(2)^10.93±0.11^*a*^	^(1)^11.77±0.15^*b*^	^(2)^8.33±0.11^*c*^	^(2)^9.13±0.11^*d*^
Chloroform	*Ficus carica*	^(3)^8.07±0.06^*a*^	^(2)^9.83±0.15^*b*^	^(3)^0.00^*c*^	^(3)^9.47±0.11^*d*^
	*Solanum incanum*	^(4)^7.17±0.06^*a*^	^(3)^8.83±0.06^*b*^	^(3)^0.00^*c*^	^(4)^10.13±0.11^*d*^
Amoxicillin	^(5)^13.10^a^	^(4)^15.10^*b*^	^(4)^11.00^c^	^(5)^12.30^*d*^	

Data are expressed as a mean of three determinations ± SD. There is a significant difference in the inhibition zones with different-letter superscripts across a row and parenthesis-enclosed-number superscripts down a column (*p* < 0.05).

**Table 7:** Inhibition zones, including well diameter, of samples against fungal growth.

Solvent	Samples	Inhibition zones (in mm) for fungi	
		*Aspergillus niger*	*Penicillium cyclopium*
Ethanol	*Ficus carica*	^(1)^8.53±0.12^*a*^	^(1)^8.47±0.12^*a*^
	*Solanum incanum*	^(2)^8.27±0.12^*a*^	^(2)^7.17±0.06^*b*^
Chloroform	*Ficus carica*	^(3)^0.00^*a*^	^(3)^10.27±0.12^*b*^
	*Solanum incanum*	^(3)^0.00^*a*^	^(3)^10.33±0.12^*b*^

Data are expressed as a mean of three determinations ± SD. There is a significant difference in the inhibition zones with different-letter superscripts across a row and parenthesis-enclosed-number superscripts down a column (*p* < 0.05).

**Table 8:** Heavy metal levels in the respective plant materials determined by ICP-OES.

Metals	Metal content in samples (mg/kg)		Permissible limit (mg/kg)	Reference
	*Ficus carica*	*Solanum incanum*		
Pb	ND	ND	10	[31]
Cr	^(1)^0.64±0.01^*a*^	^(2)^0.29±0.02^*a*^	1.5	
Cd	^(1)^0.16±0.01^*b*^	^(1)^0.12±0.01^*b*^	0.3	
Mn	^(1)^3.67±0.10^*c*^	^(2)^0.17±0.03^*b*^	200	
Cu	^(1)^0.38±0.04^*d*^	^(1)^0.33±0.04^*a,c*^	10	
Zn	ND	ND	50	
Fe	^(1)^0.62±0.07^*a*^	^(2)^0.25±0.01^*a,d*^	20	

ND: not detected. Data are expressed as a mean of three determinations ± SD. There is a significant difference in the metal contents with different-letter superscripts down a column and parenthesis-enclosed-number superscripts across a row (*p* < 0.05).

## Data Availability

The data used to support the findings of this study are included within the article.
